# Molecular Mimics of the Tumour Antigen MUC1

**DOI:** 10.1371/journal.pone.0049728

**Published:** 2012-11-14

**Authors:** Tharappel C. James, Ursula Bond

**Affiliations:** Moyne Institute for Preventive Medicine, School of Genetics and Microbiology, Trinity College, University of Dublin, Dublin, Ireland; UCLA, United States of America

## Abstract

A key requirement for the development of cancer immunotherapy is the identification of tumour-associated antigens that are differentially or exclusively expressed on the tumour and recognized by the host immune system. However, immune responses to such antigens are often muted or lacking due to the antigens being recognized as “self”, and further complicated by the tumour environment and regulation of immune cells within. In an effort to circumvent the lack of immune responses to tumour antigens, we have devised a strategy to develop potential synthetic immunogens. The strategy, termed mirror image phage display, is based on the concept of molecular mimicry as demonstrated by the idiotype/anti-idiotype paradigm in the immune system. Here as ‘proof of principle’ we have selected molecular mimics of the well-characterised tumour associated antigen, the human mucin1 protein (MUC1) from two different peptide phage display libraries. The putative mimics were compared in structure and function to that of the native antigen. Our results demonstrate that several of the mimic peptides display T-cell stimulation activity *in vitro* when presented by matured dendritic cells. The mimic peptides and the native MUC1 antigenic epitopes can cross-stimulate T-cells. The data also indicate that sequence homology and/or chemical properties to the original epitope are not the sole determining factors for the observed immunostimulatory activity of the mimic peptides.

## Introduction

Immunotherapy is currently considered an important augmentative therapy for the treatment of cancer. Several complementary treatment regimes such as immune cell transfer (adoptive immunotherapy), tumour vaccines, cytokine therapy, and monoclonal antibodies targeting immune response checkpoints [1]–[3] may form part of this therapy. One form of adoptive immunotherapy for cancer patients involves loading tumour-associated antigens (TAAs) *in vitro* onto macrocytic dendritic cell (DCs) precursors isolated from the patient. The DCs are matured *in vitro* before presenting them to the circulating lymphocytes by infusing back into the patient leading to activation of one or more subsets of T-cells. Some of the activated T-cells may become tumour-infiltrating lymphocytes (TIL), attacking and eradicating the tumour [4]. Alternatively, TILs can also be directly isolated from the patient's tumour tissue, expanded *in vitro* prior to reintroduction back to the patient [5].

A key requirement for the development of cancer immunotherapy is the identification of antigenic structures that are differentially or exclusively expressed on tumours and recognised by the host immune system. Using a variety of approaches, many TAAs have been identified and in the majority of cases, they arise from non-mutated proteins that are over-expressed in tumour cells but which are also present in normal cells [6], [7]. The failure of the immune system to respond to tumour antigens often results from a lack of immunogenicity of many TAAs as a result of their being recognised as ‘self-antigen’ or due to masking of tumour antigens from the immune system, coupled with deficiencies in immune surveillance, effectors mechanisms of immune cells and immune suppression in the tumour microenvironment [8], [9].

In an effort to circumvent the lack of immune responses to TAAs and to generate a bank of novel tumour-specific immunogenic antigens, we have devised a strategy to develop potential synthetic immunogens. It is based on the concept of molecular mimicry as demonstrated by the idiotype/anti-idiotype paradigm in the immune system. ‘Idiotype’ refers to the antibody segment that recognises the antigen epitope while the term anti-idiotype describes amino acid sequences that binds to the idiotype. Thus anti-idiotypes are ‘mirror images’ of native antigens and function as their structural mimics. Anti-idiotype molecules have been used to induce immune responses against viral, bacterial and parasitic infections and also to some cancers [10]–[16].

The strategy, termed mirror-image phage display (MIPD), involves a two-step biopanning of phage display libraries. First, a known or a suspected tumour antigen is used as a target for the biopanning of a random peptide-display bacteriophage library to identify “recogniser” peptides (analogous to idiotypes) of the target. In the second step, a synthetic “recogniser” peptide is used as the target in a ‘reverse biopanning’ experiment to identify potential mimic molecules (analogous to anti-idiotype) of the original tumour antigen. We have previously used the MIPD platform to identify molecular mimics of antigenic peptides associated with Heat Shock Proteins (HSPs-ap) in the breast tumour cell line MDA-MB-231 [17]. Despite the heterogeneous pool of the starting target (HSP-ap), we successfully deciphered the amino acid sequences of a number recogniser peptides, and used some of these as targets in reverse biopanning to identify potential mimic peptides. Some of these mimics displayed immunostimulatory properties when loaded onto dendritic cells. However, because of the heterogeneity of the starting target (HSP-ap), the relationship between the native antigen and the mimic could not be ascertained at the structural level.

Here, we set out to test the MIPD technology platform and to examine the nature of the mimic peptides, specifically their functional and/or structural similarity to the native antigen. Towards this end, we used a well-characterised tumour associated antigen, the human mucin1 protein (MUC1) as a target in the MIPD platform and compared the putative mimics in structure and function to that of the native antigen. The human MUC1 belongs to a large class of high molecular weight O-linked glycoproteins (mucins) and is expressed at the luminal membrane of most glandular and ducal epithelial cells. MUC1 is over-expressed in approximately 90% of human breast cancers [18]. It is also over-expressed in many other human tumours such as colorectal, pancreatic and ovarian cancers [19]. Over-expression is correlated with poor prognosis, increased tumor growth, invasion and metastasis [20]. Cell invasion by MUC1-expressing tumour cells can be suppressed by over-expression of the micro RNA, miR-145, which directly interacts with MUC1 mRNA in the 3′ UTR region [21]. MUC1 contains a large extracellular domain composed of a variable (20–125) number of tandem repeats (VNTR) of a 20-amino acid sequence. The extracellular domain is cleaved post-translationally from the remainder of the polypeptide but remains non-covalently associated, producing a tethered bottle-brush-like structure of heavily glycosylated VNTRs protruding up to 100 nm from the cell surface. Glycosylation of the VNTRs differs in normal and tumour cells with tumour cells generally showing a hypo-glycosylated state. It has been suggested that the hypo-glycosylated state of MUC1 in tumour cells contributes to the increased immunogenicity through damasking antigenic sites on the protein [22].

MUC1 is able to break immune tolerance and both humoral and cell-mediated immune responses against MUC1 have been detected in cancer patients [23].

Patients with breast and colorectal cancer express anti-MUC1 IgG antibodies at much higher frequencies than healthy individuals and both MHC-restricted and non-restricted T-cell responses against MUC1 have also been detected in cancer patients. The intrinsic qualitative and quantitative differences of MUC1 in tumour and non-tumour cells, together with its inherent immunogenicity, makes it particularly suitable for its development as a cancer vaccine. A large number of Phase I/II clinical trials with MUC1 derived peptides have been conducted. While promising responses such as cytotoxic T-cell responses and disease stabilization have been reported, no single approach has proved successful as a definitive therapeutic strategy. In this study, we have identified recogniser and mimic peptides to two MUC1 antigens using the MIPD technology platform. The recogniser peptides show similarities to known anti-MUC1 antibodies while some of the mimic peptides contain amino acid motifs present in the original antigenic MUC1 peptides. The mimic peptides were tested for their ability to activate lymphocytes when presented by dendritic cells *in vitro*.

## Materials and Methods

### Ethics Statement

All experiments were carried out in compliance with the Helsinki Declaration (http://www.wma.net/e/policy/b3.htm) and according to the Cruelty to Animals Act, 1876 as amended by European Communities (Amendment of Cruelty to Animals Act 1876) Regulations 2002 and 2005. Human blood samples were anonymously obtained from the Irish Blood Transfusion Board. All animal experiments were carried out under a license issued by Minister of Department of Health and Children, Government of Ireland to Dr T.C. James (Ref. B100/3897) and were approved by the Ethics Committee, Trinity College Dublin (Ref 080906).

### Cell lines and Media

The breast cancer cell line MDA-MB-231, a gift from Prof. D.J Bouchier-Hayes (Royal College of Surgeons Ireland, Surgery Department, Beaumont Hospital, Dublin, Ireland) [24], was grown in RPMI-1640 supplemented with 10% foetal calf serum (FCS; Sigma Chemical Company). The media also contained 2 mM L-glutamine, 1× antibiotic/antimycotic solution (Sigma Chemical Co.) and 100 U/mL nystatin suspension. AIM V serum-free medium for immune cell culture was obtained from Invitrogen Inc. (Life Technologies, Inc. Carlsbad, CA USA).

### Peptides

All synthetic peptides were sourced from GL Biochem Shanghai Ltd. (Shanghai, PRC). The peptides were C-terminal amidated unless otherwise specified. In some cases, additional N-terminal amino acids (shown in lower case, in the text) were added to facilitate biotinylation and/or to reduce steric hindrance during biopanning. For competitive elution as well as for DC loading immunostimulation studies, peptides without biotin or N-terminal amino acid extensions were used.

### Mirror-Image Phage Display (MIPD) Biopanning

**Figure 1:**
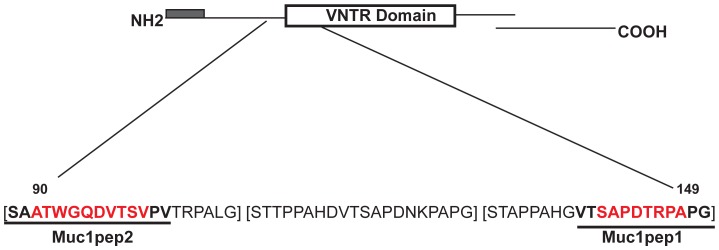
Structure of the MUC1 protein. The secretory signal peptide is shown as a shaded box at the N-terminus. The variable number tandem repeat (VNTR) is shown as an open rectangle. The small C-terminal domain, which is proteolytically derived from a common precursor protein and containing the transmembrane domain is also shown. The expanded region shows the amino acid sequences of three VNTR repeats [in brackets]. The amino acid sequences of the 12-mer and 14-mer peptides (MUC1pep1 and MUC1pep2 respectively) used for biopanning are underlined. The sequence of the core peptides used for eluting bound phage, competition ELISA and DC stimulation are shown in red.

The Ph.D. C7C library was used for the first step biopannings while both the Ph.D. C7C and the Ph.D-12 libraries (New England Biolabs Inc., MA) were employed in the reverse biopannings. The basic outline of biopanning procedure was described earlier [17], [25]. Approximately 10^11^–10^12^ phage particles from the relevant phage display library were used for each biopanning experiment. In the first step, the peptides **VTSAPDTRPAPG** (MUC1pep1) or **SAATWGQDVTSVPV** (MUC1pep2; 5 µg each; Fig. 1) were immobilised on coated wells of a 96-well Maxisorb microtitre plate (Nunc-Nalge Inc.). The blocking, binding and washing strategies were carried out as instructed by the library (Ph.D-C7C) manufacturer with modifications [17]. Phages binding specifically to the target peptides were eluted with the core peptides, (SAPDTRPA and ATWGQDVTSV respectively, shown in red in Fig. 1) at 10 µg/mL. Amplified phage stocks from this first round of biopanning were used for the second round biopanning step as follows: biotinylated peptides (5 µg each), consisting of the core peptide sequence (kSAPDTRPA or kATWGQDVTSV; Fig. 1, shown in red) and the amplified phage stocks (∼10^11^ phage particles) were incubated together in 100 µL PBS. The phage particles binding to the target peptides were recovered through the addition of Streptavidin-paramagnetic beads (PMSt.1.0; G.Kisker Biotech, Dortmund, Germany) and recovered magnetically, followed by extensive (15×1 mL each) washing and competitive elution with the corresponding core peptides as described above. Following each round of biopanning, the eluted phages were amplified to high titer according to supplier's instructions. The third round biopanning involved an *in vivo* biopanning step. A xenografted human mammary cell adenocarcinoma [26] was developed as follows: MDA-MB231 cells (10^7^ cells/injection) were embedded in Matrigel (BD Biosciences, Oxford, UK) and implanted into the mammary pads of 4-week old nu/nu mice (Hsd:Athymic Nude-Foxn1^nu^; Harlan Laboratories Inc., Indianapolis, IN). Two weeks later, 10^8^ phages amplified from the second round biopanning were injected in 0.1 mL PBS through the lateral caudal vein. Animals were sacrificed 15 minutes later and xenografted tumour (1–1.5 cm), liver and lung tissues were collected. The tissues were chopped into 1–2 mm sizes and washed extensively three times in (10 mL each) PBS and any remaining blood was drained off onto Whatman No. 1 filter paper. Following homogenization of the tissues, an aliquot of 10^8^ bacteria were added and incubated for 20 minutes at 37°C before diluting to 20 mL with L-Broth. After a further 6–8 hour incubation, to facilitate phage amplification, the lysate was centrifuged at 8000×g to pellet the bacteria and the phage in the supernatant was concentrated through Poly Ethelyne Glycol (PEG) precipitation as described by New England Biolabs. The fourth round biopanning was identical to the first round using amplified phage from the *in vivo* biopanning step. The phage particles from the final fourth round eluate were plated at low density to allow isolation of single phage clones. The DNA insert from the amplified phage clones was sequenced and the recogniser peptide sequences deduced.

To identify potential mimics to MUC1pep1 and MUC1pep2 peptides, selected recogniser peptides, N-terminally biotinylated, were synthesised and used as bait in a similar biopanning procedure as above. Special attention was taken to ensure the binding of the bait peptide to the matrix. The competitive elution of bound phages from the immobilised recogniser peptides was accomplished by eluting with the 7-mer recogniser peptide. After 4 rounds of biopanning, the eluted phage displaying mimic peptides were analysed as above.

### M13 phage ELISA

Biotinylated recogniser peptides (200 pmoles) were bound to streptavidin-coated Maxisorb immunolon Nunc 96-well plates. Following blocking with 0.5% BSA and 0.1 mM d-biotin in PBS, the plates were incubated for three hours with selected M13 phage clones (10^6^ pfu) displaying the mimic peptides. Unbound phages were removed by repeated washing with excess PBS containing 0.05% TWEEN-20 (PBS-Tween). The amount of phage bound to the immobilised peptide was detected and quantified by incubation with HRP conjugated anti-M13 monoclonal antibody (anti-M13-HRP; Amersham Biosciences, UK; 1∶2,500) and the premade chromogenic substrate TMB (Sigma Chemical.Co., St. Louis, MO. USA) according to suppliers instructions.

For competition ELISA, the basic setup was the same as above with the exception that the mimic phages and the competing original Muc1 core peptides (MUC1pep1 or MUC1pep2; Fig. 1 sequences shown in red) were incubated together before the mixture was applied to the immobilized peptide.

### Isolation of monocytes and lymphocytes and generation of immature DCs

Buffy coat packs from healthy female donors were obtained from the Irish Blood Transfusion Service, Dublin-8. PBMCs were prepared by Leucosep pre-filled density gradient centrifugation media (Greiner Bio One Ltd., UK). Monocytes were isolated by positive selection of CD14^+^ cells using anti-CD14 microbeads (Miltenyi Biotec, Bergisch Gladbach, Germany). The unbound CD14- fraction, which mainly consist of lymphocytes (B-, T-, NK- and NKT-cells) were cryopreserved for later use as responder cells.

Immature DCs (iDCs) were generated by culturing the CD14^+^ monocytes for 6–8 days in AIM V serum-free medium (Life Technologies Co. Carlsbad, USA) supplemented with 60 ng/mL recombinant human granulocyte macrophage colony stimulating factor (GM-CSF; PeProtech Inc., Rocky Hill, NJ, USA)) and 150 ng/mL recombinant human IL-4 (PeProtech Inc.). Medium and cytokines were replaced every 2 days.

### *In vitro* stimulation assay of lymphocytes

Lymphocyte stimulation was performed using a modification of a published procedure [27]. Approximately 10^4^ immature dendritic cells (iDCs) were incubated with 150 µl of AIM-V medium containing 10 µg/mL lipopolysaccharide (LPS) or 10 ng/mL Tumour Necrosis Factor-α (TNF-α) and peptides (25 µg/mL), or media alone (negative control). After 24 hour incubation, the cells were pelleted and the culture media were assayed for IL-12 by capture ELISA (see below). The iDCs were washed free of all peptides and co-stimulants and resuspended in fresh AIM-V medium. The antigen loaded iDCs were incubated with CD14^−^ PBMCs at a ratio of 1∶10 (DCs to PBMCs) in a final volume of 150 µL AIM-V medium. This is referred to as ‘first stimulation’. After 2 days incubation the culture supernatants (100 µL) were removed and replaced with 100 µL fresh media (supplemented with human recombinant IL-2 (25 ng/mL)). This was repeated every second day for 8 days. These cells are designated as ‘primed CD14^−^ PBMCs. The spent media was assayed for IFN-γ content by capture ELISA. On day 6, a new batch of matured DCs from the same donor was incubated with different peptides as before but without LPS or TNF-α. After 48 hrs, the culture supernatants were assayed for IL-12 levels as before. Following the washes, these loaded DCs were incubated with the ‘primed CD14^−^ PBMCs’ (either in the same or a different combination, e.g., MUC1pep1 primed CD14^−^ PBMCs’ in second stimulation with MP1) at a ratio of 1∶10 (iDC∶CD14^−^ PBMCs) and with media changes every 3 days.

### Measurement of cytokine release

The IFN-γ released by the stimulated CD14^−^PBMC was measured by ELISA using capture and detection antibody pairs (PeProtech Rocky Hill, NJ USA). Similarly, IL-12 production by iDCs was detected using either the ELISA kit also purchased from PeProtech or the DuoSet human IL-12p40 antibodies (R&D Systems, Minneapolis, MN). The diluted capture antibody (50 µL) for IFN-γ or IL-12 were immobilised on Nunc Immunolon Maxisorb 96-well plates. Following washing in PBS-Tween, the plates were blocked and 50 µL culture media were assayed as prescribed by the antibody manufacturers.

## Results

The 20-amino acid peptide [STAPPAHGVTSAPDTRPAPG]_20–125_, in the VNTR is the major immunogenic determinant of MUC1 (Fig. 1). The most frequent epitopes recognised by human MUC1 antibodies include the peptides RPAPGS, PPAHGVT and PDTRP within the VNTR [28]–[32] (Fig. 1). Antigenic epitopes have also been identified in MUC1 in regions outside of the core VNTR repeat region [33]. One such epitope spans amino acids 90–110 in the extracellular domain (Fig. 1). Sequence analysis indicates that this region contains a degenerate VNTR sharing 10 of the 20 amino acids of the core repeat. This peptide sequence was identified as a potential HLA-A2 binding peptide and was capable of activating T-cells as measured by IFN-γ production [34].

As a first step in MIPD, to generate ‘recogniser’ peptides of MUC1, two synthetic peptides (Fig. 1, underlined sequences), **VTSAPDTRPAPG** (MUC1pep1) encompassing the major epitope(s) within the VNTR repeat and the 14-mer peptide **SAATWGQDVTSVPV** (MUC1pep2) corresponding to amino acid 90–104 region of the degenerate-VNTR epitope, and sharing a common motif VTS with MUC1pep1, were chemically synthesised and used as targets in the biopanning of a C7C M13-phage display library. The first two rounds of biopanning were carried out *in vitro* [17], as outlined in the Methods section. In order to select for phage that specifically ‘home’ to MUC1-bearing tumours, the amplified phage from round two were injected into a tumour bearing nu/nu mouse xenografted with human breast cancer cell line MDA-MB-231. The tumour was excised and any bound phages were recovered as described in the Methods section. The extracted phages were used in a further round of biopanning to corresponding MUC1 peptides *in vitro*. Following the fourth round of selection, the peptides displayed on the selected phages were deciphered from nucleic acid sequencing data (Table 1). In addition to recovering phages from the engraft breast tumour in round three, phages were also recovered from liver and lung tissue and used separately in biopanning against MUC1 peptides in the fourth round. These phages neither bore any resemblance to those identified from the xenografted tumour tissue nor were they enriched in any specific sequence (data not shown).

**Table 1:** MUC1 peptides and their corresponding recogniser peptides.

Target MUC1 Peptides	Recogniser Peptides (frequency)	Major anti-MUC1 CDRs*
VTSAPDTRPAPG (Mucpep1)	**HSQ**LPQV (RP1)	RS**SQ**TIV**HS**NGKIYLE (L-CDR1)
	PHETP**HQ** (RP2)	**HQ**WSSSPRT (L-CDR3)
	DPQV**NP**A (RP3)	YISFDGSNNY**NP**SLKN (H-CDR2)
SAATWGQDVTSVPV (Mucpep2)	TNTL**SNN** (RP4)	EIRLK**SNN**FATHFAESVKG (H-CDR2) YISFDG**SNN**YNPSLKN (H-CDR2) RSSQTIVH**SN**GKIYLE (L-CDR1)
	P**GS**EHKH (RP5)	YISFD**GS**NNYNPSLKN (H-CDR2) FQ**GS**HVPWT (L-CDR3)
	QNDRH**PR** (RP6)	HQWSSS**PR**T (L-CDR3)
	H**AT**RHTT (RP7)	**AT**SNLAS (L-CDR2)

* From [35] Sequence similarities between anti-MUC1 CDRs and recognisers are shown in bold and underlined.

Since the peptides are identified through direct peptide-peptide interaction between the baits (MUC1pep(n)) and the displayed sequences, they were named “MUC1 recogniser” peptides (MUC1RP). Three MUC1RPs were isolated from biopanning with the MUC1pep1 (Table 1). Two of these [RP1 and RP3] share the common motif PQV. With the exception of a preponderance of histidine and proline residues, there appears to be no other amino acid commonalities between the three RPs derived from MUC1pep1. Furthermore the three RPs differ in their degree of hydrophobicity, overall charge and isoelectric points (Table 2). Interestingly, the RPs share amino acid motifs previously identified in the ‘complementarity determining regions’ (CDRs; notably in the CDR1 and CDR3 domains) of an anti-MUC1 VNTR antibody (Table 1, panel 3, relevant amino acids underlined and bolded) [35].

**Table 2:** Physical properties of recogniser peptides.

Peptide	Hydrophobicity (%)	Hydrophilicity (%)	Charge	Isoelectric point	Attribute
RP1	43	14	+1	7.55	Basic
RP2	29	43	+1	6.5	Basic
RP3	57	14	−1	3.75	Acidic
RP4	14	0	0	6.02	Neutral
RP5	14	57	+2	7.72	Basic
RP6	14	57	+2	10.45	Basic
RP7	14	43	+3	10.55	Basic

The four recogniser peptides [RP4-RP7] derived from MUC1pep2 share no common amino acid motifs between them, however three of them (RP5-7) are hydrophilic in nature, basic and positively charged (Table 2). RP4 on the other hand is a relatively neutral peptide with no hydrophilic residues and contains only a single hydrophobic amino acid. Similar to the RPs derived from MUC1pep1 (RP1-3), the MUC1pep2 recognisers (RP4-7) also contain motifs previously identified in the CDRs of MUC1 antibody [35]. In particular the motif SNN in RP4 is present in the heavy chain-CDR2 (H-CDR2). An SN motif has also been identified in a light chain-CDR1 (L-CDR1) region of the anti-MUC antibody. The motifs GS, PR and AT present in RP5, RP6 and RP7 respectively have also been identified within anti-MUC CDR regions (Table 1, relevant amino acids underlined and bolded).

Two of the recogniser phages (RP1 and RP4; recognisers of MUC1pep1 and MUC1pep2 respectively) were chosen for the reverse biopanning step to identify potential mimics of these MUC1 peptides. Synthetic peptides corresponding to RP1 and RP4 were generated and used as targets for biopanning. Two different phage display libraries were used, the first being the C7C library described above. This library displays random 7-mer peptides constrained between two cysteine residues on the PIII minor coat protein. The second library, PhD-12, displays unconstrained random 12-mer peptides also fused to the PIII protein. Following four rounds of biopanning, the sequences displayed on the surfaces of the selected phages were identified (Table 3). The RP1 peptide (recogniser of MUC1pep1) yielded one 7-mer (MP1) and two linear 12-mer (MP2 and MP3) putative mimic peptides. One of these, MP3, shares the tri-peptide sequence motif TRP, within MUC1pep1 (Table 3). The TRP motif has been identified as a major antigenic determinant within the MUC1 VNTR region [28]. Similarly, the MP2 mimic peptide shares the motif RxAP with the native MUC1pep1. Note that the AP di-peptide motif is repeated three times in the original MUC1 VNTR and twice in the MUC1pep1 target peptide used in the first step biopanning (see Fig. 1). The only mimic selected by RP1 from the 7-mer C7C library is MP1 (Table 3), which did not share any obvious tri- or di-peptide blocks with either of the native MUC1 peptide (MUC1pep1). The RP4 peptide (recogniser of MUC1pep2) when used in MIPD selected two 7-mer peptides (MP4 and MP5) as putative mimics of MUC1pep2. Like MP1, MP4 and MP5 also do not show any obvious sequence similarity to MUC1pep2 (Table 3). A comparison of various physical and chemical properties of the MP2 and MP3 peptides reveal that they display a high degree of hydrophobicity similar to that of the native MUC1pep1 (Table 4). In contrast, the putative mimic peptide MP1 is less hydrophobic and net charge neutral. The two 7-mer peptides (MP4 and MP5) selected as putative mimics of MUC1pep2 are hydrophilic (Table 4). MP4 is slightly basic while MP5 is charge neutral. This is in contrast with the native MUC1pep2, which is a hydrophobic acidic peptide.

**Table 3:** MUC1 peptides, corresponding recognisers and putative mimic peptides.

MUC1 peptide	Recogniser Peptide	Mimic Peptide
VTSAPD**TRPAP**G (MUC1pep1)	HSQLPQV (RP1)	cNTQTLGMc (MP1)
		IVI**R**Q**AP**SHELI (MP2)
		LPPNEF**TRP**SVD (MP3)
SAATWGQDVTSVPV (MUC1pep2)	TNTLSNN (RP4)	cNDRSHSGc (MP4)
		cKEDARQAc (MP5)

Sequence similarities between mimic peptides (MP2 and MP3) and MUC1pep1 are shown in bold and underlined.

**Table 4:** Physical properties of MUC1pep1, MUC1pep2 and their putative mimic peptides MP1-5.

Peptide	Hydrophobicity (%)	Hydrophilicity (%)	Charge	Isoelectric Point	Attribute
MUC1pep1	46	15	0	6.23	Neutral
MP1	25	0	0	6.02	Neutral
MP2	58	25	+1	7.55	Basic
MP3	50	25	−1	4.19	Acidic
MUC1pep2	40	10	−1	3.75	Acidic
MP4	0	38	+1	7.37	Basic
MP5	25	50	0	6.23	Neutral

### Interaction of native, recogniser and mimic peptides

**Figure 2:**
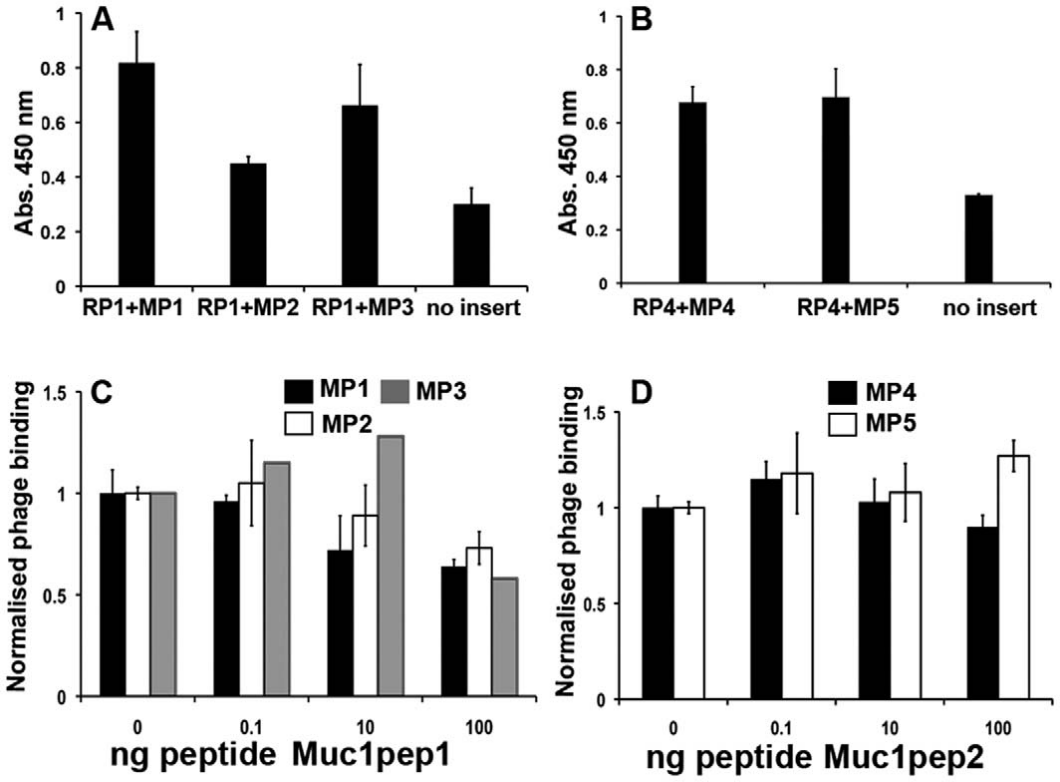
Interaction of Recogniser and Mimic peptides. **A.** Phages bearing the mimic peptides MP1-3 or no insert were incubated with biotinylated RP1 peptide, immobilized on Streptavidin coated microtitre plates. Bound phages were quantified by measuring OD_450 nm_ following incubation with HRP conjugated anti-M13 monoclonal antibody and the chromogenic substrate TMB. Error bars represent the standard deviation between two independent samples. **B.** as for A except that the bound peptide RP4 was incubated with the phages bearing the peptides MP4-5 or no insert. **C.** As in **A** except that the phage particles were pre-incubated with increasing concentrations (ng) of the MUC1pep1 core peptide prior to incubation with RP1. Phage binding in the presence of competitor was normalised to the binding in the absence of competitor, which was set at an arbitrary value of 1.0. Error bars represent the normalized % error from two independent samples. MP1; black, MP2, white, MP3, grey. **D.** as in **B** except that the immobilized peptide was RP4 which was incubated with the mimic peptides MP4 (black) or MP5 (white). The competing peptide was the MUC1pep2 core peptide.

To examine the binding affinity of recogniser peptides for their corresponding mimics, ELISA assays were carried out. Typically, biotinylated forms of the recogniser peptides, immobilised on microtitre wells, were incubated with a fixed amount of phage particles displaying the corresponding mimic peptides Phage binding to the immobilised peptide was quantified as described in Materials and Methods. As shown in Fig. 2A and B, phage particles displaying the mimic peptides (MP1-5) show binding to their corresponding recogniser peptide (RP1 or RP4). In all cases the binding was statistically significant over the binding observed with phage particles lacking a displayed peptide (no insert).

To examine the relative affinity of the recogniser peptides for the mimic peptides and the original MUC1pep from which they were derived, mimic phages were pre-incubated with or without increasing concentrations of the corresponding native MUC1pep1 or MUC1pep2 prior to incubation with the bound recognizer peptides. As shown in Fig. 2C, increasing concentrations of MUCpep1 reduces the binding of the corresponding mimic peptides (MP1-3) to the recogniser peptide RP1. In contrast, similar concentrations of MUCpep2 did not reduce the binding of MP4 or MP5 to RP4 (Fig. 2D), suggesting that RP4 has a higher affinity for the mimic peptides than for the original MUC1pep2.

### Immunostimulatory Activity of MUC1 Mimic Peptides

**Figure 3:**
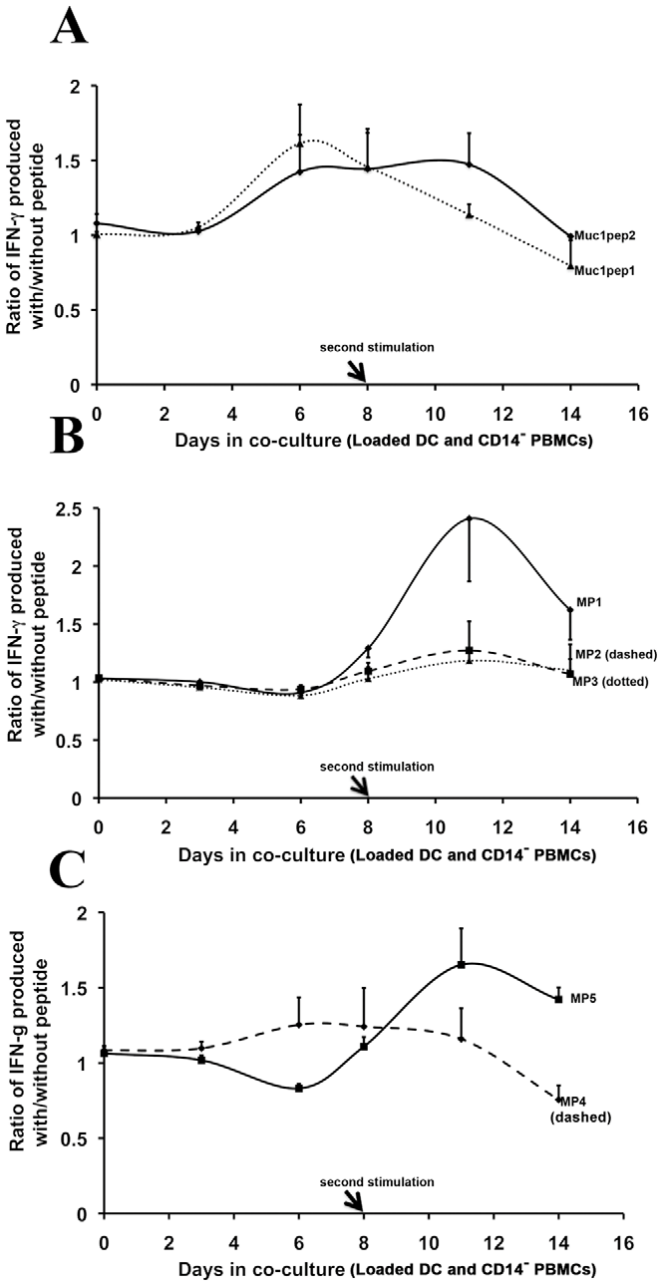
Immunostimulation of CD14- cells with peptide-loaded DCs. **A.** CD14^−^ cells were co-cultured with DCs loaded with MUC1pep1 core peptide (dotted line) or MUC1pep2 core peptide (solid line). After 8 days (arrow), the cells were re-stimulated by addition of a fresh batch of autologous DCs. T-cell stimulation was monitored by quantifying the amount of IFN-γ released into the medium. The IFN-γ levels were normalized to the levels produced following incubation with DCs that had no peptides loaded. Error bars represent the normalized % error from two independent experiments. **B.** As in A except that DCs were loaded with MP1 (solid line), MP2 (dashed line) or MP3 (dotted line). **C.** As in A except that DCs were loaded with MP4 (dashed line) and MP5 (solid line).

We next set out to test the immunostimulatory potential of the MUC1 mimic peptides *in vitro*. Briefly, matured DCs were loaded with either the original Muc1pep1 or MUC1pep2 core peptides (Fig. 1) or their putative mimic peptides. As a negative control DCs were left unloaded or loaded with an unrelated peptide, the yeast α-mating type factor peptide, (α-factor). The loaded and unloaded DCs were incubated with autologous CD14^−^ cells (T-cell pool) that had been isolated from the same PBMC pool. Since previous studies have shown that CD14^−^ cells often require multiple rounds of activation by DCs to invoke stimulation and IFN-γ release, the same CD14^−^ cells were subjected to a second round of stimulation on day 8 with a fresh batch of DCs loaded with the same peptide as before. T-cell stimulation was measured by quantification of interferon-γ (IFN-γ) released to the medium during the first (days 0–8) and second stimulation (days 9–14) as described in the Methods and Materials section. Based on our hypothesis, true mimic peptides (MPs) should stimulate as effectively as their corresponding native MUC1pep(n) counterparts. As shown in Figure 3A, maximum IFN-γ release from CD14^−^ cells was observed on day 6 in cells incubated with MUC1pep1 and MUC1pep2. At its peak, the level of IFN-γ produced approximated 630–645 pg/mL for both peptides. No additional IFN-γ release was achieved by a second stimulation with a fresh batch of MUC1pep1-loaded DCs on Day 8 (Fig. 3A, dotted line). In contrast, IFN-γ release persisted up to day 13 in CD14^−^ cells following the second stimulation with fresh DCs loaded with MUC1pep2 (Fig. 3A, solid line), maintaining levels as high as 633 pg/mL on day 11. The ability of DCs-loaded with the mimic peptide to stimulate CD14^−^ cells was next examined. Of the mimic peptides, MP1 (Fig. 3B, solid line) and MP5 (Fig. 3C, solid line) produced the highest level of IFN-γ release by CD14^−^ PBMCs with maximum levels generated on day 11, after the second round of stimulation with DCs. A modest level of stimulation (20–30%) was also achieved with DCs loaded with the peptides MP2 (Fig. 3B, dashed line), MP3 (Fig. 3B, dotted line) and MP4 (Fig. 3C, dashed line). At their peak (day 11), following the second stimulation on day 8, the mimic peptides MP1 and MP5 induced interferon levels up to 700 pg/mL, which is in the same range at that achieved following stimulation with MUC1pep1 and MUC1pep2-loaded DCs. In all cases, the absolute IFN-γ levels showed a steady decline by day 14 (data not shown).

No stimulation was observed when DCs were loaded with the negative control peptide (α-factor) (data not shown) or when DCs were used without any loaded peptide. A similar pattern of T-cell stimulation was observed in a second independent experiment using healthy immune cells from a different blood donor (data not shown). Taken together, the data indicates that a subset of the mimic peptides display immunostimulatory activity *in vitro* and induce IFN-γ levels similar to that achieved with the native MUC1 antigens, however at least two rounds of stimulation are required to elicit IFN-γ release.

### Cross-stimulation of mimic and MUC peptides

**Figure 4:**
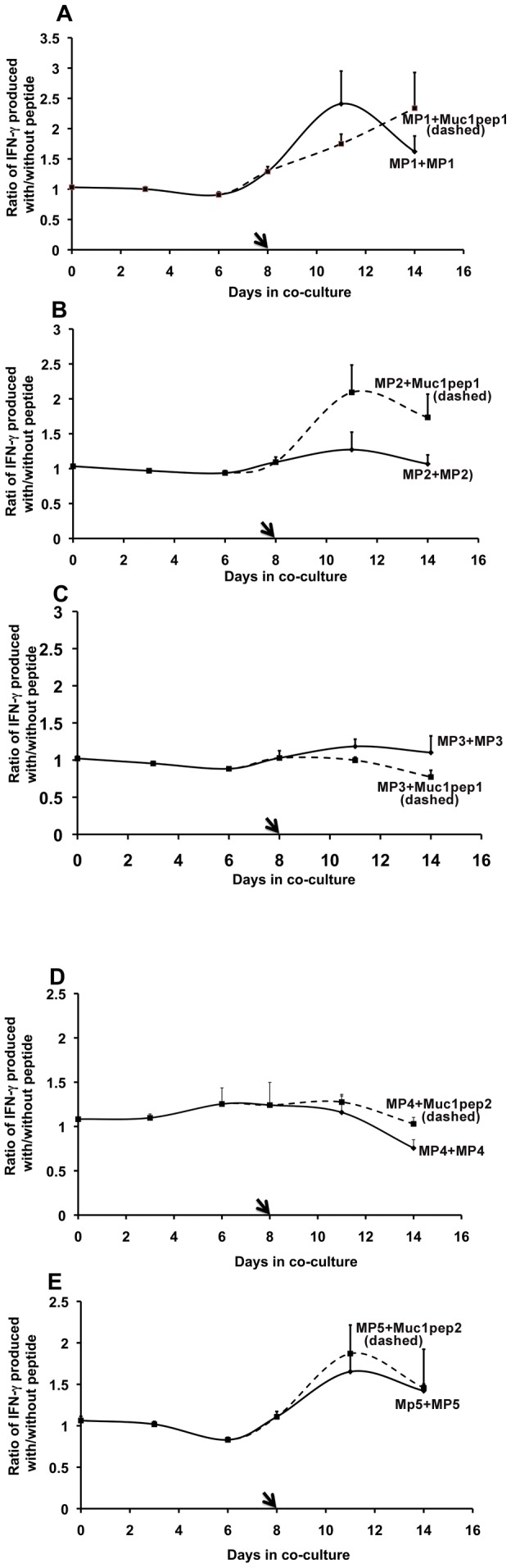
Cross-stimulation of CD14- cells by Mimic and native MUC1 peptides. CD14^−^ cells were incubated with DCs loaded with mimic peptides and then restimulated with a fresh batch of DCs loaded with either the same peptide or the original MUC1 core peptides (Fig. 1). T-cell stimulation was monitored by quantifying the amount of IFN-γ released into the medium. The IFN-γ levels were normalized to the levels produced following incubation with DCs that had no peptides loaded. Error bars represent the normalized % error from two independent experiments. **A.** CD14^−^ cells stimulated with MP1 and then re-stimulated on day 8 with MP1 (solid line) or cross-stimulated with MUC1pep1 (dashed line). **B.** CD14^−^ cells stimulated with MP2 and then re-stimulated on day 8 with MP2 (solid line) or cross-stimulated with MUC1pep1 (dashed line). **C.** CD14^−^ cells stimulated with MP3 and on day 8, re-stimulated with MP3 (solid line) or cross-stimulated with MUC1pep1 (dashed line). **D.** CD14^−^ cells stimulated with MP4 and on day 8 re-stimulated with MP4 (solid line) or cross-stimulated with MUC1pep2 (dashed line). E. CD14^−^ cells stimulated with MP5 and on day 8, re-stimulated with MP5 (solid line) or cross-stimulated with MUC1pep1 (dashed line).

We hypothesised that mimic peptides identified using the mirror-image phage display platform should possess biological properties similar to the original native peptide. Therefore, we tested to see if cross-stimulation of CD14^−^ cells could be achieved by the mimic and MUC1 peptides using the consecutive stimulation approach described above. First, CD14^−^ cells were incubated with DCs loaded with the mimic peptides. After 8 days, the cells were re-stimulated by addition of a fresh batch of DCs loaded with either the same mimic peptide (as for Fig. 3) or the corresponding original MUC1pep(n), peptides. IFN-γ release was monitored over the course of the incubation. Secondary stimulation by MUC1pep1 was observed in CD14^−^ cells previously stimulated with DCs loaded with MP1 and MP2 but not with MP3 (Fig. 4A–C; dashed lines). The level of IFN-γ release was similar following a secondary stimulation with either MP1 or MUC1pep1, although the timeline of stimulation differed in each case (Fig. 4A). Secondary stimulation with MUC1pep1 substantially increased the IFN-γ released by cells treated with MP2-loaded DCs (Fig. 4B; dashed line) compared to that achieved by secondary stimulation by the autologous peptide alone. No further increase in IFN-γ release was observed in CD14^−^ stimulated with MP3-loaded DCs when re-stimulated with MUC1pep1-loaded DCs (Fig. 4C, dashed lines). Secondary stimulation with MUC1pep2-loaded DCs enhanced IFN-γ output of CD14^−^ cells previously stimulated with MP5-DCs (Fig. 4E; dashed line) but not with MP4-DCs (Fig. 4D; dashed line).

**Figure 5:**
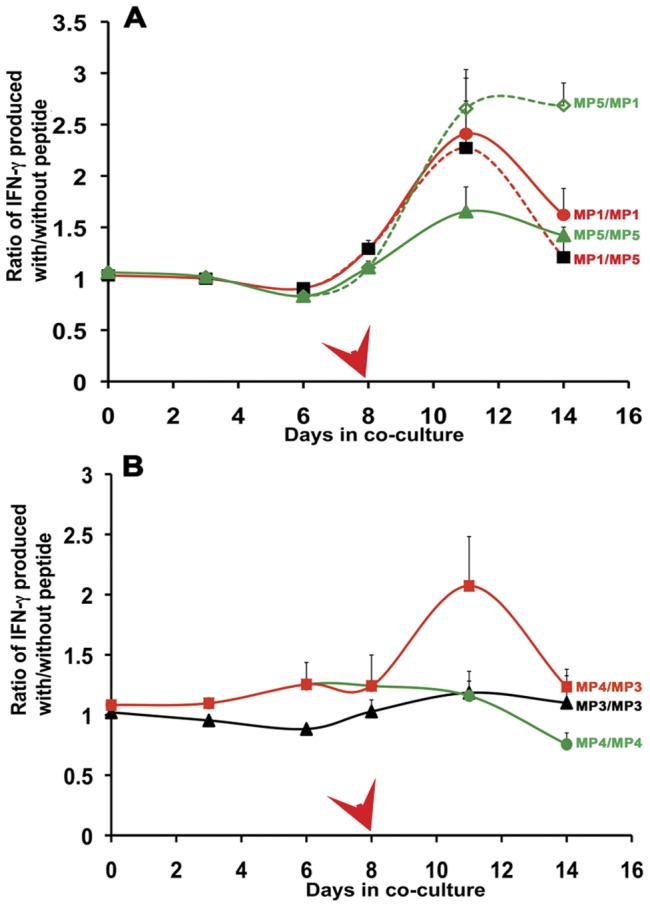
Cross-stimulation of CD14- cells by mimic peptides. CD14^−^ cells were incubated with DCs loaded with mimic peptides and then re-stimulated with a fresh batch of DCs loaded with same or a different mimic peptide. T-cell stimulation was monitored by quantifying the amount of IFN-γ released into the medium. The IFN-γ levels were normalized to the levels produced following incubation with DCs that had no peptides loaded. Error bars represent the normalized % error from two independent experiments. The combination of peptides are indicated in the figure and are as follows **A.** MP1 followed by MP1; red solid line (•), MP1 followed by MP5; red dashed line (▪), MP5 followed by MP5; green solid line (▴) and MP5 followed by MP1; green dashed line (⋄). **B.** MP3 followed by MP3; black solid line (▴), MP4 followed by MP4; green solid line (•), MP4 followed by MP3; red solid line (▪).

We also examined the ability of the mimic peptides to cross-stimulate. We observed cross-stimulation between the peptides MP1 and MP5 regardless of the order in which the peptides were used for stimulation of CD14^−^ cells (Fig. 5A; green and red dashed lines). Likewise cross stimulation was observed between peptides MP4 and MP3 (Fig. 5B; red solid line) compared to that achieved by secondary stimulation with DCs loaded either of the autologous peptides (Fig. 5B, green and black lines). Other combinations of mimic peptides did not show such cross-stimulatory activity (data not shown).

## Discussion

### MUC is a suitable antigen for adoptive immunotherapy

The identification of suitable tumour antigens for cancer vaccine development is a priority goal in cancer research. The Translational Research Working group of the National Cancer Institute has recently developed a set of criteria for the assessment and prioritizing of potential cancer vaccine targets. Based on properties such as immunogenicity, tumour specificity, cellular localisation and expression levels, and percentage of patients with antigen positive cancer, MUC1 ranked second for prioritisation in a list of 75 previously characterised tumour antigens [36]. To date, several clinical trials have been conducted to examine immune responses to MUC1-based antigens. While both humoral and cell mediated responses have been observed in patients with various forms of cancers, neither tumour elimination nor satisfactory tumour regression has been achieved [23], [37]. Several clinical trials using modified MUC1 antigens are currently ongoing and a successful outcome may not be far away.

The lack of progress in the development of a MUC1-based cancer vaccine may stem from some of the inherent properties of MUC1 protein such as variations in glycosylation patterns in tumour and non-tumour cells. Many vaccine trials were based on the non-glycosylated MUC1 VNTR peptide as the antigen source, however even a fully glycosylated MUC1 polypeptide elicited only modest antibody responses [38], [39]. One recent study using a tripartite vaccine comprised of a glycosylated form of MUC1 repeat peptide, SAPDT(αGalNAc)RPAP, conjugated to a murine MHC class II restricted T-helper epitope and a potent agonist of the Toll-like receptor 2(Pam3CysSK4) was shown to elicit both T lymphocyte and antibody-dependent cell-mediated cytotoxic responses in a mouse mammary model [29]. Likewise a vaccine based on a glycosylated form of the VNTR repeat conjugated to a tetanus toxoid displayed a strong and selective immune response [40]. Thus, combinations of immune modulators and antigenic peptides may provide more potent anti-tumour responses.

### MIPD Strategy

Here, we have attempted an alternative approach for the design of potential immunostimulatory antigenic peptides specific for a given tumour. The approach is based broadly on the concept of idiotype/anti-idiotype antibodies. Being mimics of the original antigen, anti-idiotype antibodies can invoke prolonged immune responses and have been used as cancer vaccines [41]–[43]. Anti-idiotype antigens are considered to have merits over native antigens particularly as they may be presented to the immune system in a different molecular and biological context.

Here, rather than using antibody-based molecules, we used a strategy termed “mirror image phage display” (MIPD) to identify potential mimics of known MUC1 antigenic peptides. The amino acid sequences in the CDRs of anti-MUC1 antibodies and their anti-idiotype antibodies have been identified previously [35]. Thus we can compare the MIPD derived mimic peptides with that of the conventionally characterised anti-idiotype sequences. As with idiotype/anti-idiotype antibodies, we envisaged that the MIPD identified mimic peptides will truly “mimic” the original antigen in their ability to recognize and bind to a common intermediary molecule, termed here as “recognisers”. Analysis of the sequences within the recogniser peptides, generated from two different MUC1 peptides, MUC1pep1 from within the VNTR and MUC1pep2 from a degenerate VNTR, revealed some common motifs previously found within the CDRs of the anti-MUC1 antibody AR20.5 [35].

The recogniser peptides derived from MUC1pep1 were more hydrophobic in nature than those derived from MUC1pep2. Thus, the characteristics of the original peptide influences the types of peptides obtained in the biopanning process. The congruence of the anti-MUC1pep1-antibody CDRs and the MUC1RPs at the amino acid level re-enforces the view that phage display technology has the potential to identify the true antigen-antibody interacting domains.

During the biopanning for MUC1 recognisers, we included a single round of *in vivo* biopanning to the MUC1-expressing breast cancer tumour MDA-MB-231, which had been xenografted into a nu/nu mouse. This *in vivo* biopanning step significantly enriched for recogniser peptides with similarity to the conventionally identified idiotypes. Biopanning with phage extracted from liver or lung tissue from the same xenografted mouse against MUC1 peptides produced a non-overlapping set of peptides that bore no relationship to original idiotypes.

Peptides RP1 and RP4 were chosen for mimic generation. Here we used two phage display libraries, one displaying 7-mer constraint peptide and the second displaying a linear 12-mer peptide. The derived mimics, in particular, those obtained from the 12-mer unconstraint library, contained motifs present in the original MUC1 peptides. For example, MP3 contained the tri-peptide TRP, which is present in one of the major epitopes derived from MUC1pep1 VNTR [28]– while the peptide motif RxAP found in MP2 is also a major MUC1 epitope. Interestingly, the anti-idiotype antibody AR42.1 derived from the idiotype sequences of the anti-MUC1 monoclonal antibody AR20.5, contained the sequence GPLYRPGEGY in H-CDR3, which shows homology (x**P**xx**RP**xx**G**x) to sequences within the core repeat vtsa**P**dt**RP**ap**G**s, suggestive of the molecular mimicry of anti-idiotype antibodies. While the homologies detected at the sequence level are present in the mimic phage isolates from the 12-mer (Ph.D. 12) library, the mimic phages isolated from the cysteine constrained 7-mer library did not bear any overt sequence homology to either of the original MUC1 peptides. Analysis of the physical properties of the peptides did not reveal any striking patterns, however as observed for the recogniser peptides, those derived from MUC1pep1 appeared to be more hydrophobic in nature while as before, the MUC1pep2 derived peptides displayed hydrophilic characteristics.

In general, antigenic peptides tend to be hydrophobic in nature and epitope/antibody interaction favors hydrophobic interfaces; however, the antigenicity of the mimic peptides cannot be solely deduced from their chemical properties. To test antigenicity directly, *in vitro* immune-stimulation experiments were performed with DCs loaded with the original MUC1 peptides and also with the newly identified mimic peptides. The mimic peptides, when presented by DC cells, displayed varying abilities to stimulate CD14^−^ cells to release IFN-γ. By far, the highest level of stimulation was achieved with the 7-mer peptides MP1 and MP5 while a lower but sustained stimulation was also achieved with MP4. Much lower levels of CD14^−^ stimulation was achieved with the 12-mer peptides MP2 and MP3 despite these peptides containing amino acid motifs found in the original MUC1 peptides. The level of CD14^−^ stimulation did not correlate with the degree of hydrophobicity of the peptides, the only commonality being that the best stimulators were neutral in charge and/or were constrained by di-sulphide bridges. These findings could inform research into epitope design for other tumour antigens.

### Cross-Stimulation of Mimic and MUC1 peptides

Previously, using heat-shock protein 70-associated antigenic peptides (HSP70-ap) and their mimics as potential immunotherapeutic tumour antigens, we have shown that T-cells can be stimulated either with DCs loaded with HSP70-ap or with its putative mimic peptide [17]. A second stimulation of the same T-cells with a fresh batch of antigen or its mimic loaded DCs yielded a secondary response from the T-cells as measured by IFN-γ production. Based on our hypothesis, we would predict that if the mimic peptides bear structural or biological properties similar to the original MUC1 peptides then they should enhance a primary stimulation by the original peptides and vice versa. We found that secondary stimulation with the native Muc1 peptides could be achieved for a sub-set of the mimic peptides. In particular, IFN- γ release was increased two-fold when CD14^−^ cells, first stimulated with MP2, were re-challenged with DCs loaded with MUC1pep1, the response being better than that of either of the peptides alone (Fig. 4). Likewise, cross-stimulation was observed between MP1 and MUC1pep1 and between MP5 and MUC1pep2. This cross-stimulation may be due to amplification of the original primed T-cell pool or primary stimulation of a separate sub-set of T-cells. Our results favor the former as the increased response was observed only when specific combinations of peptides were used for first and second stimulation. Likewise, cross stimulation was achieved with various combinations of mimic peptides, in particular MP3/MP4 and MP1/MP5. We note that the cross-stimulation occurred with mimics derived from different original MUC1 peptides. This cross-stimulation may arise from the common motifs in the original MUC1 peptides, or to similar tertiary structures present in the two peptides. This is not surprising since both MUC1pep1 and MUC1pep2 are related by ancestry to the same repeat motif of the VNTR. Taken together, the data indicates that mimics of known antigens can be generated by the relatively straightforward high throughput mirror-image phage display biopanning technique. The availability of idiotype and anti-idiotype sequences for the VNTR repeat in question facilitated the recogniser phage selection process. Similarly, the *in vivo* biopanning step narrowed selection of recogniser phages binding to tumour-specific molecules. The derived mimics are capable of stimulating T-cells to release IFN-γ and some bear cross-stimulatory activity with the original antigens. The comparison of immuno-stimulatory activity of the mimics derived from different phage display libraries suggests that constrained 7-mer peptides are superior to linear 12-mer peptides in inducing T-cell proliferation and that sequence homology and/or chemical properties to the original epitope are not determining factors for immunostimulatory activity of mimic peptides.

The synthetic mimics of MUC1, described here, may be capable of directing T-cell responses against tumour cells expressing MUC1, leading to tumour cell killing. The mimic peptides may also be capable of eliciting B-cell responses against MUC1, although we have not tested this directly here. Immune responses could be enhanced by conjugation of the mimic peptides to adjuvants such as tetanus toxoid, Toll-like receptor agonists or by association with immune carrier proteins or lipid immunostimulants [40], [44]. The mimic peptides displaying the highest levels of T-cell responses did not resemble the original MUC1 antigens at the primary amino acid sequence level. Such peptides are less likely to be recognized as “self” by the immune system and may therefore avoid immune surveillance mechanisms. Since MUC1 is over expressed in several types of cancers and its over-expression is correlated with poor prognosis [20] and increased cell invasion and metastasis [21], vaccination with the mimic peptides may therefore be useful in preventing metastasis of MUC1-overexpression tumours.

Finally, the technology described here could be used to generate mimic peptides to the large number of characterised tumour-associated antigens. Thus our findings may inform the design of other tumour antigens for the development of cancer vaccines.

## References

[1] Caux C, Zitvogel L (2012) Recent successes of cancer immunotherapy: a new dimension in personalized medicine? Target Oncol 7: 1–2. 22350488 10.1007/s11523-012-0211-3

[2] Whiteside TL (2012) Disarming suppressor cells to improve immunotherapy. Cancer Immunol Immunother 61: 283–288. 22146892 10.1007/s00262-011-1171-7 PMC11028463

[3] Wicherek L, Jozwicki W, Windorbska W, Roszkowski K, Lukaszewska E, et al (2011) Analysis of Treg cell population alterations in the peripheral blood of patients treated surgically for ovarian cancer - a preliminary report. Am J Reprod Immunol 66: 444–450. 21624000 10.1111/j.1600-0897.2011.01024.x

[4] Bray SM, Vujanovic L, Butterfield LH (2011) Dendritic cell-based vaccines positively impact natural killer and regulatory T cells in hepatocellular carcinoma patients. Clin Dev Immunol 2011: 249281. 21969837 10.1155/2011/249281 PMC3182577

[5] Shirakura Y, Mizuno Y, Wang L, Imai N, Amaike C, et al (2012) T-cell receptor gene therapy targeting melanoma-associated antigen-A4 inhibits human tumor growth in non-obese diabetic/SCID/gammacnull mice. Cancer Sci 103: 17–25. 21951605 10.1111/j.1349-7006.2011.02111.x PMC11164177

[6] Renkvist N, Castelli C, Robbins PF, Parmiani G (2001) A listing of human tumor antigens recognized by T cells. Cancer Immunol Immunother 50: 3–15. 11315507 10.1007/s002620000169 PMC11036832

[7] Mellman I, Coukos G, Dranoff G (2011) Cancer immunotherapy comes of age. Nature 480: 480–489. 22193102 10.1038/nature10673 PMC3967235

[8] Hurwitz AA, Watkins SK (2012) Immune suppression in the tumor microenvironment: a role for dendritic cell-mediated tolerization of T cells. Cancer Immunol Immunother 61: 289–293. 22237887 10.1007/s00262-011-1181-5 PMC6948839

[9] Hammerstrom AE, Cauley DH, Atkinson BJ, Sharma P (2011) Cancer immunotherapy: sipuleucel-T and beyond. Pharmacotherapy 31: 813–828. 21923608 10.1592/phco.31.8.813 PMC4159742

[10] Nisonoff A, Lamoyi E (1981) Implications of the presence of an internal image of the antigen in anti-idiotypic antibodies: possible application to vaccine production. Clin Immunol Immunopathol 21: 397–406. 6173154 10.1016/0090-1229(81)90228-2

[11] Raychaudhuri S, Saeki Y, Fuji H, Kohler H (1986) Tumor-specific idiotype vaccines. I. Generation and characterization of internal image tumor antigen. J Immunol 137: 1743–1749. 3018080

[12] Raychaudhuri S, Saeki Y, Syma, Kohler H (1986) Method and means for selection of anti-idiotype antibodies and their use for the diagnosis, monitoring, treatment and/or prevention of cancer, autoimmune or infectious disease. WIPO Patent Application WO/1989/005309.

[13] Kennedy RC, Dreesman GR, Kohler H (1985) Vaccines Utilizing Internal Image Anti-idiotypic Antibodies that Mimic Antigens of Infectious Organisms. Biotechniques 3: 404–410.

[14] Kennedy RC, Eichberg JW, Dreesman GR (1986) Lack of genetic restriction by a potential anti-idiotype vaccine for type B viral hepatitis. Virology 148: 369–374. 3484564 10.1016/0042-6822(86)90333-8

[15] Herlyn D, Ross AH, Koprowski H (1986) Anti-idiotypic antibodies bear the internal image of a human tumor antigen. Science 232: 100–102. 3952496 10.1126/science.3952496

[16] Elaine D, Dorothee H, Hilary K, Kevin R, Tadeusz W (1985) Immune response to tumours and viruses induced by anti-idiotype antibodies European Patent Organisation EP0141783 (A2) ― 1985-05-15.

[17] Arnaiz B, Madrigal-Estebas L, Todryk S, James TC, Doherty DG, et al (2006) A novel method to identify and characterise peptide mimotopes of heat shock protein 70-associated antigens. J Immune Based Ther Vaccines 4: 2. 16603084 10.1186/1476-8518-4-2 PMC1482705

[18] Lacunza E, Baudis M, Colussi AG, Segal-Eiras A, Croce MV, et al (2010) MUC1 oncogene amplification correlates with protein overexpression in invasive breast carcinoma cells. Cancer Genet Cytogenet 201: 102–110. 20682394 10.1016/j.cancergencyto.2010.05.015

[19] Singh AP, Chaturvedi P, Batra SK (2007) Emerging roles of MUC4 in cancer: a novel target for diagnosis and therapy. Cancer Res 67: 433–436. 17234748 10.1158/0008-5472.CAN-06-3114

[20] Baldus SE, Monig SP, Huxel S, Landsberg S, Hanisch FG, et al (2004) MUC1 and nuclear beta-catenin are coexpressed at the invasion front of colorectal carcinomas and are both correlated with tumor prognosis. Clin Cancer Res 10: 2790–2796. 15102686 10.1158/1078-0432.ccr-03-0163

[21] Sachdeva M, Mo YY (2010) MicroRNA-145 suppresses cell invasion and metastasis by directly targeting mucin 1. Cancer Res 70: 378–387. 19996288 10.1158/0008-5472.CAN-09-2021 PMC2805032

[22] Hiltbold EM, Vlad AM, Ciborowski P, Watkins SC, Finn OJ (2000) The mechanism of unresponsiveness to circulating tumor antigen MUC1 is a block in intracellular sorting and processing by dendritic cells. J Immunol 165: 3730–3741. 11034378 10.4049/jimmunol.165.7.3730

[23] Tang CK, Katsara M, Apostolopoulos V (2008) Strategies used for MUC1 immunotherapy: human clinical studies. Expert Rev Vaccines 7: 963–975. 18767946 10.1586/14760584.7.7.963

[24] Pidgeon GP, Barr MP, Harmey JH, Foley DA, Bouchier-Hayes DJ (2001) Vascular endothelial growth factor (VEGF) upregulates BCL-2 and inhibits apoptosis in human and murine mammary adenocarcinoma cells. Br J Cancer 85: 273–278. 11461089 10.1054/bjoc.2001.1876 PMC2364032

[25] Siebke C, James TC, Cummins R, O'Grady T, Kay E, et al (2012) Phage display biopanning identifies the translation initiation and elongation factors (IF1alpha-3 and eIF-3) as components of Hsp70-peptide complexes in breast tumour cells. Cell Stress Chaperones 17: 145–156. 22002548 10.1007/s12192-011-0295-1 PMC3273561

[26] Al-Hajj M, Wicha MS, Benito-Hernandez A, Morrison SJ, Clarke MF (2003) Prospective identification of tumorigenic breast cancer cells. Proc Natl Acad Sci U S A 100: 3983–3988. 12629218 10.1073/pnas.0530291100 PMC153034

[27] Fleischer K, Schmidt B, Kastenmuller W, Busch DH, Drexler I, et al (2004) Melanoma-reactive class I-restricted cytotoxic T cell clones are stimulated by dendritic cells loaded with synthetic peptides, but fail to respond to dendritic cells pulsed with melanoma-derived heat shock proteins in vitro. J Immunol 172: 162–169. 14688322 10.4049/jimmunol.172.1.162

[28] Thie H, Toleikis L, Li J, von Wasielewski R, Bastert G, et al (2011) Rise and fall of an anti-MUC1 specific antibody. PLoS One 6: e15921. 21264246 10.1371/journal.pone.0015921 PMC3021526

[29] Lakshminarayanan V, Thompson P, Wolfert MA, Buskas T, Bradley JM, et al (2012) Immune recognition of tumor-associated mucin MUC1 is achieved by a fully synthetic aberrantly glycosylated MUC1 tripartite vaccine. Proc Natl Acad Sci U S A 109: 261–266. 22171012 10.1073/pnas.1115166109 PMC3252914

[30] Blixt O, Bueti D, Burford B, Allen D, Julien S, et al (2011) Autoantibodies to aberrantly glycosylated MUC1 in early stage breast cancer are associated with a better prognosis. Breast Cancer Res 13: R25. 21385452 10.1186/bcr2841 PMC3219186

[31] Graves CR, Robertson JF, Murray A, Price MR, Chapman CJ (2005) Malignancy-induced autoimmunity to MUC1: initial antibody characterization. J Pept Res 66: 357–363. 16316451 10.1111/j.1399-3011.2005.00297.x

[32] Quinlin IS, Burnside JS, Dombrowski KE, Phillips CA, Dolby N, et al (2007) Context of MUC1 epitope: immunogenicity. Oncol Rep 17: 453–456. 17203187 10.3892/or.17.2.453

[33] Heukamp LC, van der Burg SH, Drijfhout JW, Melief CJ, Taylor-Papadimitriou J, et al (2001) Identification of three non-VNTR MUC1-derived HLA-A*0201-restricted T-cell epitopes that induce protective anti-tumor immunity in HLA-A2/K(b)-transgenic mice. Int J Cancer 91: 385–392. 11169964 10.1002/1097-0215(200002)9999:9999<::aid-ijc1051>3.0.co;2-z

[34] Tsang KY, Palena C, Gulley J, Arlen P, Schlom J (2004) A human cytotoxic T-lymphocyte epitope and its agonist epitope from the nonvariable number of tandem repeat sequence of MUC-1. Clin Cancer Res 10: 2139–2149. 15041735 10.1158/1078-0432.ccr-1011-03

[35] Luo D, Qi W, Ma J, Wang YJ, Wishart D (2000) Molecular mimicry of human tumor antigen by heavy chain CDR3 sequence of the anti-idiotypic antibody. J Biochem 128: 345–347. 10965030 10.1093/oxfordjournals.jbchem.a022759

[36] Cheever MA, Allison JP, Ferris AS, Finn OJ, Hastings BM, et al (2009) The prioritization of cancer antigens: a national cancer institute pilot project for the acceleration of translational research. Clin Cancer Res 15: 5323–5337. 19723653 10.1158/1078-0432.CCR-09-0737 PMC5779623

[37] Rong Y, Qin X, Jin D, Lou W, Wu L, et al (2011) A phase I pilot trial of MUC1-peptide-pulsed dendritic cells in the treatment of advanced pancreatic cancer. Clin Exp Med 10.1007/s10238-011-0159-0 21932124

[38] Sorensen AL, Reis CA, Tarp MA, Mandel U, Ramachandran K, et al (2006) Chemoenzymatically synthesized multimeric Tn/STn MUC1 glycopeptides elicit cancer-specific anti-MUC1 antibody responses and override tolerance. Glycobiology 16: 96–107. 16207894 10.1093/glycob/cwj044

[39] Ryan SO, Turner MS, Gariepy J, Finn OJ (2010) Tumor antigen epitopes interpreted by the immune system as self or abnormal-self differentially affect cancer vaccine responses. Cancer Res 70: 5788–5796. 20587526 10.1158/0008-5472.CAN-09-4519 PMC2905500

[40] Kaiser A, Gaidzik N, Westerlind U, Kowalczyk D, Hobel A, et al (2009) A synthetic vaccine consisting of a tumor-associated sialyl-T(N)-MUC1 tandem-repeat glycopeptide and tetanus toxoid: induction of a strong and highly selective immune response. Angew Chem Int Ed Engl 48: 7551–7555. 19685547 10.1002/anie.200902564

[41] Yuan W, Xia G, Zhao C, Sui C, Ma J (2012) Anti-idiotypic single chain mimicking CA125 linked with tuftsin provides protective immunity against ovarian cancer in mice. Mol Med Report 5: 388–394. 10.3892/mmr.2011.643 22020296

[42] Wilkinson RW, Ross EL, Lee-MacAry AE, Laylor R, Burchell J, et al (2000) A transgenic mouse model for tumour immunotherapy: induction of an anti-idiotype response to human MUC1. Br J Cancer 83: 1202–1208. 11027434 10.1054/bjoc.2000.1431 PMC2363579

[43] Mitchell MS (2002) Cancer vaccines, a critical review–Part II. Curr Opin Investig Drugs 3: 150–158. 12054066

[44] Westerlind U, Kunz H (2011) Synthetic vaccines from tumor-associated glycopeptide antigens. Chimia (Aarau) 65: 30–34. 21469441 10.2533/chimia.2011.30

